# Vetiver Essential Oil in Cosmetics: What Is New?

**DOI:** 10.3390/medicines4020041

**Published:** 2017-06-16

**Authors:** Pauline Burger, Anne Landreau, Marie Watson, Laurent Janci, Viviane Cassisa, Marie Kempf, Stéphane Azoulay, Xavier Fernandez

**Affiliations:** 1 Université Côte d’Azur, CNRS, ICN, Nice CEDEX 2, 06108 Parc Valrose, France; Pauline.BURGER@unice.fr (P.B.); Anne.LANDREAU@unice.fr (A.L.); Stephane.AZOULAY@unice.fr (S.A.); 2 Université d’Angers, UFR Santé, 16 Boulevard Daviers, CEDEX 01, 49045 Angers, France; 3 Extraits de Bourbon, 2 rue Maxime Rivière, La Réunion, 97490 Ste Clothilde, France; marie.watson81@gmail.com (M.W.); ljanci974@gmail.com (L.J.); 4 Laboratoire de Bactériologie-Hygiène, Centre Hospitalier Universitaire, 4 rue Larrey, CEDEX 09, 49933 Angers, France; vivianecassisa@yahoo.fr (V.C.); MaKempf@chu-angers.fr (M.K.)

**Keywords:** *Vetiveria zizanioides* (L.) Nash, essential oil composition, cosmetic bioassays, *Candida* spp., antifungal activities, antibacterial activities

## Abstract

**Background:** Vetiver is a key ingredient for the perfume industry nowadays. However, with the constant and rapid changes of personal tastes, this appeal could vanish and this sector could decline quite quickly. New dissemination paths need to be found to tap this valuable resource. **Methods:** In this way, its potential use in cosmetics either as an active ingredient per se (with cosmeceutical significance or presenting antimicrobial activity) has hence been explored in vitro. **Results:** In this contribution, we demonstrated that vetiver essential oil displays no particularly significant and innovative cosmetic potential value in formulations apart from its scent already largely exploited. However, evaluated against twenty bacterial strains and two *Candida* species using the in vitro microbroth dilution method, vetiver oil demonstrated notably some outstanding activities against Gram-positive strains and against one *Candida glabrata* strain. **Conclusions:** Based on these findings, vetiver essential oil appears to be an appropriate aspirant for the development of an antimicrobial agent for medicinal purposes and for the development of a cosmetic ingredient used for its scent and displaying antimicrobial activity as an added value.

## 1. Introduction

*Chrysopogon zizanioides* (L.) Roberty (formerly known as *Vetiveria zizanioides* (Linn.) Nash), commonly known as vetiver is a perennial bunchy herbaceous species of the Poaceae family that develops at altitudes up to 2000 m in almost every soil type, although well-drained sand is considered the most appropriate subsoil for its growth [1,2]. The stems being stiff, vetiver tufted grass can attain up to 2 m height [2]. Native to India, vetiver was disseminated around the world some 100 years ago and is since widely cultivated in tropical regions for many different purposes (current major producers include Haiti, India, Indonesia, and Reunion Island) [2,3].

Almost all parts of vetiver are exploited in traditional medicines: reported to be among others, carminative, diuretic, diaphoretic and emmenagogue, vetiver also constitutes a renowned parasitic and anthelmintic agent [1]. Vetiver is considered in several folk medicines as an alexiteric agent, e.g., a preservative against poisons and venoms: a paste of fresh roots is advocated against snakebites and scorpion stings [1]. In Trinidad, vetiver tea is used to treat notably flu, colic, nausea and pleurisy [4]. In the Philippines and Thailand, the root’s decoction is ingested to dissolve gallstones [5]. An infusion prepared from pulverized roots provides a refreshing drink recommended to cool fevers and cure stomach diseases [2,6]. Vetiver preparations are recommended topically to relieve pains in case of skin burns [2]. Topically applied leaves paste was reported to relieve rheumatisms, lumbagos and sprains [1]. Stem decoction is also used by tribes of West Bengal to relief urinary tract infection [1].

Because the substantial vertical root system of vetiver is finely structured and very strong (often measuring more than 3 m), this species is highly drought-tolerant and can help to stabilize soils, protecting them against sheet erosion [2,3]. Extremely resistant to pests and diseases, the plant also protects fields against vermin and other pests, as well as weeds [2]. The multipurpose species is also use as phytoremediation agent for metal-contaminated soils, mattress stuffing, animal bedding, animal feed, mulch (used for weed control in coffee, cocoa and tea plantations), a food additive/flavoring agent, etc. [7,8,9], but is mainly cultivated today to produce essential oil (EO). Obtained from the distillation of dried roots, vetiver EO is highly valued for cosmetics (soaps, deodorants, etc.), aromatherapy, perfumes (as base notes as well as perfume fixative), etc. [3]. There are two main vetiver forms: one fertile form produced in India (generally known a khus oil) and one non-seeding, sterile form grown outside India; both produce essential oil [3]. To favor the essential oil production, the plant needs to grow in humid to sub-humid conditions [2]. Vetiver EO displays pleasant heavy, earthy-woody extremely persistent notes that could not be reproduced synthetically because of the EO’s molecular complexity [10,11,12]: highly appreciated for its tenacity, it enters the formula of numerous modern fragrances both feminine and masculine [11,13]. Vetiver oil has furthermore been extensively used in modern aromatherapy, notably for its sedative qualities and for its balancing/regulatory activity on skin [5].

In this way, vetiver oil constitutes a key ingredient for the perfume/cosmetic industry, but with the constant and rapid changes of personal tastes, this general appeal could decline quite quickly and new dissemination paths need to be identified to tap this valuable resource. In fact, the current annual trade estimated around 250 tons EO worldwide, worth $20–200 million per year, clearly demonstrates this good’s importance [14]. Haiti is the world’s first exporter of vetiver and more than 50,000 families are totally and directly dependent on this resource for their living [15]. A careful examination of the literature demonstrates that if the chemical composition of vetiver essential oil has been extensively analyzed, its biological properties have only been poorly investigated. Furthermore, given the heterogeneity of the results of such analysis, a report must be drawn up and complementary analysis are necessary to assess vetiver EO’s bioactivities. This article hence reports the investigation of vetiver oil’s potential use in cosmetics as an active ingredient per se (with cosmeceutical significance, e.g., with whitening, antioxidant, anti-aging and anti-inflammatory potencies, or presenting antimicrobial activity) to circumvent an eventual disinterest for this fragrance by the public and consequently the perfume industry and to enlarge even more its exploitation range.

## 2. Materials and Methods

All chemicals were obtained from Sigma-Aldrich (St. Quentin Fallavier, Auvergne-Rhône-Alpes, France) unless otherwise stated.

### 2.1. Essential Oil

Commercial vetiver essential oils (Ve1501, Ve1502 and Ve1503) were acquired from the CAHEB (Coopérative Agricole des Huiles Essentielles de Bourbon), i.e., the agricultural cooperative for essential oils of Reunion Island. Essential oils were solubilized in dichloromethane at a concentration of 80 mg/mL for both the GC/FID and GC-MS analyses. For the cosmetic bioassays, EOs were diluted at a concentration of 3.433 mg/mL in DMSO to reach a sample’s final concentration per well of 100 µg/mL.

### 2.2. Olfactory Analysis

Static olfactory evaluation was carried out by a trained perfumer (Iris Consulting, Grasse, France); to this end, the various vetiver EOs were prepared at 10% in ethanol 96%.

### 2.3. GC/FID Analyses

The GC/FID analyses were performed using an Agilent 6890N gas chromatograph equipped with a FID, an electronic pressure control injector (Agilent 55 Technologies, J&W Scientific Products, Palo Alto, CA, USA), and an apolar HP-1 capillary column (100% polydimethylpolysiloxane; 0.2 mm × 50 m; film thickness, 0.33 μm). The oven temperature was programmed to rise from 40 °C to 220 °C at 2 °C/min, then increased to 270 °C at 20 °C/min and, finally, held isothermally at 270 °C for 20 min. The injector temperature was set at 250 °C and the detector temperature at 280 °C. Split ratio was 1/100 with an injection volume of 1 μL. Helium (carrier gas) was used in constant flow mode at 1 mL/min. Samples were injected in triplicate for quantification.

### 2.4. GC-MS Analyses

The GC-MS analyses were performed using an Agilent 6890 gas chromatograph (Palo Alto, CA, USA) equipped with an Agilent MSD5973N mass selective detector, a multifunction automatic sampler (Combi-Pal, CTC Analytics, Zwingen, Swiss) on an HP-1 MS capillary column (100% polydimethylpolysiloxane; 0.2 mm × 50 m; film thickness, 0.33 μm). Samples (1 µL) were injected in split mode (split ratio: 1/100) and the injector was set at a temperature of 250 °C. The carrier gas was helium in constant flow mode at 1 mL/min. The oven temperature was programmed to rise from 40 °C to 220 °C at 2 °C/min, then from 220 °C to 270 °C at 20 °C/min and kept isothermally at 270 °C for 20 min. Acquisition was performed in scan mode (40–400 a.m.u. (atomic mass unit)/s; scan rate: 3.5 scans/s) and mass spectra were generated at 70 eV.

Compound identifications were based on comparison of mass spectra with literature, commercial libraries (Wiley, NIST) and laboratory MS libraries built up from pure substances, combined with comparison of GC linear retention index (LRI) [16,17,18]. Retention indices were determined with a series of linear alkanes C6-C28 used as a reference.

### 2.5. Cosmetic Bioassays

#### 2.5.1. Materials

Untreated 96-well plates were obtained from Thermo Nunc (Thermo Fisher Scientific, Waltham, MA USA), whereas the UV-transparent ones were purchased from Costar (Thermo Fisher Scientific, Waltham, MA USA). During incubation, the 96-well plates were sealed with adhesive films (Greiner Bio-One, Courtaboeuf, Île-de-France, France). Samples for biological activity testing were prepared in 1.5 mL Eppendorf tubes, appropriate for the use of the automated pipetting system epMotion® 5075 (Eppendorf, Montesson, Île-de-France, France).

#### 2.5.2. Instrumentation

An automated pipetting system Eppendorf epMotion® 5075 was used for the biological activity assays. Absorbance measurements were performed using a microplate reader (Spectramax Plus 384, Molecular devices, Wokingham, Berkshire, UK). Data were acquired with the SoftMaxPro software (Molecular devices, Wokingham, Berkshire, UK) and inhibition percentages were calculated with the Prism software (GraphPad Software, La Jolla, CA, USA). Unless otherwise stated, the results are presented as inhibition percentages (%) calculated as follows:

I% = [(OD control − OD sample)/OD control] × 100 (with OD stating for optical density).

Similarly, all OD were corrected with the blank measurement corresponding to the absorbance of the sample before addition of the substrate, unless otherwise stated.

#### 2.5.3. DPPH Radical Scavenging Assay

The antioxidant activity of the essential oils was assessed using the DPPH (1,1-diphenyl-2-picrylhydrazyl) radical scavenging assay according to the method previously described to examine plant extracts’ antioxidant activities [19,20]: 150 µL of a solution of ethanol/acetate buffer 0.1 M pH = 5.4 (50/50) are distributed in each well, together with 7.5 µL of essential oil. Trolox (3607.8 µM in DMSO) was used as the positive control; DMSO alone was used as the negative control (OD_control_). A first optical density reading is performed at 517 nm (OD_blank_). Then, 100 µL of a DPPH solution (386.25 µM in ethanol) are distributed in each well. The plate is sealed and incubated in the absence of light at room temperature (RT). After 30 min, the final OD reading is performed at 517 nm to assess the percentage of inhibition.

#### 2.5.4. Tyrosinase Assay

Tyrosinase, a copper-containing oxidase controlling the production of the natural pigment melanin, is mainly involved in the initial rate-limiting reactions in melanogenesis: the hydroxylation of L-tyrosine into L-DOPA and its further oxidation to dopaquinone. Since this enzyme plays a key role in melanogenesis, tyrosinase inhibitors are of great concern in the development of skin whitening agents [21]. The assays are performed as follows: 150 µL of a solution of mushroom tyrosinase prepared at a concentration of 171.66 U/mL in phosphate buffer (100 mM, pH = 6.8) are distributed in each well (enzyme’s final concentration per well: 100 U/mL (monophenolic activity assay) or 50 U/mL (diphenolic activity assay)), together with 7.5 µL of essential oil. Kojic acid (3.433 mM in DMSO) is used as the positive control; DMSO alone is used as the negative control (OD_control_). The plate is filmed and incubated at RT for 20 min. Then, 100 µL of a solution of L-tyrosine (monophenolic activity assay) or L-DOPA (diphenolic activity assay) 1 mM in phosphate buffer pH = 6.8 (substrate’s final concentration per well: 0.388 mM) are distributed in each well. After 20 min of incubation, OD reading was performed at 480 nm to assess the percentage of inhibition.

#### 2.5.5. Lipoxygenase Assay

Lipoxygenase, an iron-containing enzyme catalyzing the deoxygenation of polyunsaturated fatty acids into the corresponding hydroperoxides, plays a major share in the inflammation process [22]. The assays are performed as follows: 150 µL of a solution of soybean lipoxygenase prepared at a concentration of 686.66 U/mL in phosphate buffer (50 mM pH = 8) are distributed in each well (enzyme’s final concentration per well: 400 U/mL), together with 7.5 µL of essential oil. Quercetin hydrate (1000 µM in DMSO) is used as the positive control; DMSO alone was used as the negative control (OD_control_). The plate was sealed and after 10 min of incubation, a first OD reading is performed at 235 nm. Then, 100 µL of a solution of linoleic acid prepared in phosphate buffer pH = 8 were distributed in each well. After 50 min of incubation, OD reading was performed at 235 nm to assess the percentage of inhibition.

#### 2.5.6. Elastase Assay

Elastase is a serine protease that preferentially digests elastin, a highly elastic protein working together with collagen to give skin its shape and firmness [23]. The assays are performed as follows: 150 µL of a solution of porcine pancreatic elastase prepared at a concentration of 0.171 U/mL in Tris buffer (50 mM pH = 8) are distributed in each well (enzyme’s final concentration per well: 0.1 U/mL), together with 7.5 µL of essential oil. Quercetin hydrate (8583.33 µM in DMSO) was used as the positive control: 7.5 µL were distributed per well (quercetin hydrate’s final concentration per well: 250 µM). The plate is filmed and incubated at RT for 20 min. A first OD reading is performed at 410 nm. Then, 100 µL of a solution of N-succinyl-Ala-Ala-Ala-p-nitroanilide 2.06 mM in Tris buffer (Suc-AAA-pNA’s final concentration per well: 0.8 mM) are distributed in each well. After 40 min incubation, OD reading is performed at 410 nm to assess the percentage of inhibition.

#### 2.5.7. Hyaluronidase Assay

Hyaluronidases constitute a family of enzymes that degrade hyaluronic acid, a high-molecular-weight glycosaminoglycan of the extracellular matrix. Presenting a unique ability to fix and retain H_2_O molecules, this macromolecule is widely distributed in the body and notably at the periphery of collagen and elastin fibers: it therefore plays a significant role in skin aging [24,25]. The assays are performed as follows: 150 µL of a solution of hyaluronidase prepared at a concentration of 13.3 U/mL in buffer (pH = 7) are distributed in each well, together with 7.5 µL of essential oil. Tannic acid (0.435 mg/mL in DMSO) is used as the positive control. The plate is filmed and incubated at 37 °C for 20 min. A first OD reading is performed at 405 nm. Then, 100 µL of a solution of hyaluronic acid prepared at a concentration of 150 µg/mL in buffer (pH = 5.35) are distributed in each well. After 30 min incubation at 37 °C, 50 µL of CTAB (cetyltrimethylammonium bromide) prepared at a concentration of 40 mM in a NaOH solution (2%) are added in each well and OD reading is performed at 405 nm to assess the percentage of inhibition (OD_sample_).

The results are presented as inhibition percentages (%) calculated as follows:

I% = [OD_sample_/(OD_blank_ − OD_neg.control_)]×100 (1)

#### 2.5.8. Collagenase Assay

The fibrous molecule of collagen is responsible for skin tensile strength and firmness, and constitutes therefore one of the structural units of the extracellular matrix. Collagenases are enzymes that cleave the collagen molecule within its helical region and that are more generally involved in the degradation of extracellular matrix components, thus leading to skin wrinkling [26]. The assays are performed as follows: 150 µL of a solution of collagenase prepared at a concentration of 53 U/mL in tricine buffer (pH = 7.5) are distributed in each well, together with 7.5 µL of essential oil. Tannic acid (1.72 µM in DMSO) is used as the positive control. The plate is filmed and incubated at RT for 15 min. A first OD reading is performed at 345 nm. Then, 100 µL of a solution of FALGPA (2-furanacryloyl-L-leucylglycyl-L-prolyl-L-alanine) prepared at a concentration of 5.15 mM in tricine buffer are distributed in each well. After 30 min incubation, final OD reading is performed at 345 nm to assess the percentage of inhibition (OD_sample_).

The results are presented as inhibition percentages (%) calculated as follows:

I% = [OD_sample_/(OD_blank_ − OD_neg.control_)]×100 (2)

### 2.6. Antimicrobial Bioassays

#### 2.6.1. Microorganisms

The vetiver EO’s antifungal activity was assayed on two strains of human pathogenic fungi, *Candida albicans* (ATCC 66396) and *Candida glabrata* (LMA 90-1085), obtained from the parasitology and mycology laboratory of the University Hospital of Angers, France. Molds were cultivated 2 days at 37 °C on yeast extract-peptone-dextrose agar (YPDA) containing 0.5 g/L chloramphenicol.

The vetiver EO’s bacteriostatic activity was evaluated on 20 bacterial strains obtained from the laboratory of bacteriology from the University Hospital of Angers, France: three *Acinetobacter baumannii* strains (RCH, SAN008 and AYE), four *Staphylococcus aureus* (two methicillin susceptible and two methicillin resistant clinical isolates), one *Staphylococcus epidermidis* resistant to methicillin, three *Pseudomonas aeruginosa* (ATCC27853 and two clinical isolates), two *Escherichia coli* (ATCC25922, and one clinical isolate), two *Enterobacter aerogenes* and two *Klebsiella pneumoniae* clinical isolates, one *Bacillus* sp. isolate, one *B. subtilis* isolate, and one *Corynebacterium striatum* clinical isolate. 

#### 2.6.2. Determination of Antifungal Activity 

Tests were performed following the guidelines of the reference method generally adopted to test the susceptibility of yeasts to antifungal agents [27]. Briefly, the yeast suspensions were prepared in RPMI-1640 culture medium and adjusted spectrophotometrically at 630 nm to reach a final concentration of ca. 0.5 × 10^3^ to 2.5 × 10^3^ cells per mL. Tests were performed using sterile 96-flat shaped wells microtiter plates. Serial two-fold drug dilutions were realized in DMSO: 5 μL of each essential oil was dispensed in triplicate into the wells to obtain final concentrations ranging from 250 mg/mL to 25 μg/mL. After incubation for 48 h at 37 °C, spectrophotometric MIC (minimum inhibitory concentration) endpoint was calculated from the turbidimetric data as the lowest drug concentration giving rise to a fungal growth inhibition equal to or greater than 80% of that of the drug-free control (MIC_80_). Amphotericin B, a common antifungal agent was used as the positive control to evaluate the anti-candidal efficacy of the essential oil and DMSO as the negative control.

#### 2.6.3. Determination of Bacteriostatic Activity

Tests were performed using the methodology described in the CASFM (Comité de l’Antibiogramme de la Société Française de Microbiologie) and the EUCAST (European Committee on Antimicrobial Susceptibility Testing) guidelines [28,29]. Each essential oil was diluted at 40% in DMSO under sterile conditions. Two-fold dilutions were then prepared and added to 20 mL Mueller Hinton agar (Merck, Darmstadt, Germany) before being transferred into Petri dishes: hence, EOs were tested at concentrations ranging respectively from 500 µg/mL to 2000 µg/mL (Ve1501 and Ve1502), and from 500 µg/mL to 1000 µg/mL (Ve1503). Each bacterial strain was suspended in sterile NaCl (0.15 M) and approximately 10^4^ unity forming colony per spot were inoculated on different Petri dishes using the multipoint inoculator (AQS, England). After incubation for 24 h at 37°C, MICs expressed in µg/mL were determined for tested EOs against each bacterial strain as the lowest concentration leading to bacterial growth inhibition. Control Petri dishes with no EO were also inoculated before and after each evaluation.

## 3. Results

### 3.1. Organoleptic Evaluation

An organoleptic static evaluation of the three vetiver EOs was performed by a perfumer. The essential oil Ve1501 presented a typicity loss, with weak woody and plastic notes. This typicity loss might be due to its low amount of khusimol (0.6%, Table 1), which has been characterized as partly responsible for the typical vetiver scent [30]. Ve1502 displayed smoky, gaiac-like, round peanut notes. Ve1503 appeared more characteristic and well balanced, with moderately smoky and peanut notes.

### 3.2. GC-MS Analysis

The analysis of three commercial vetiver EOs from the Reunion Island using a combination of GC-FID and GC/MS revealed that they possessed typical vetiver EOs chemical profiles, consistent with those previously published in the literature [3,31,32,33,34]. Major vetiver components have been detected, among which α-vetivone (8.4–13.3%), khusimol (0.6–8.9%), β-vetivenene (identified only in Ve1501: 4.6%), β-vetivone (2.2–3.7%), khusian-2-ol (1.8–2.3%) and khusimone (1.2–2.3%) have been characterized based on their retention indexes and mass fragmentation, as well as based on data published previously (Table 1). Trace compounds are not indicated in Table 1.

The chemical examination of vetiver essential oil has been the subject of many investigations [31,32,34]; it is therefore well known that it is one if not the most complex essential oil produced [31] and not all the components have yet been identified [3,30]. Added to this, vetiver oils display a strong compositional variability according to their geographic origins [32]. A recent review by Belhassen et al. (2015) lists the structures of the volatiles components characterized in vetiver EOs [35]. Gas chromatography constitutes the most powerful method of analysis of vetiver EOs, however preliminary efficient fractionation is generally recommended for extensive chemical characterization. Such a detailed chemical examination is not the scope of the present article (for more compositional data see [31]); the GC analyses have been performed to certify that these three samples are similar vetiver EOs of a single origin.

In their comparative study of commercial EOs from several geographical origins, Champagnat et al. (2006) observed the opposite predominance in vetiver EOs from Reunion Island [32]: khusimol is the major component (13.3%), followed by the α-vetivone (6.4%) [5]. Khusimyl acetate has also been identified in these EOs (0.4–0.7%): Andersen (1970) stated that the occurrence of esters in vetiver EOs is quite occasional and that it is generally limited to khusimyl acetate which could stem from chemical degradation in older samples [34].

### 3.3. Potential Uses of Vetiver Oils

As already stated, vetiver essential oil is a key ingredient for the perfume/personal care industry, but with the constant and rapid changes of personal tastes, this appeal could vanish quite quickly. Based on a literature survey on vetiver bioactivities and as new dissemination paths need to be found to tap this resource, the potential use of vetiver essential oil in cosmetics either as an active ingredient per se (with cosmeceutical significance or presenting antimicrobial activity) or as a natural antimicrobial agent has hence been explored in vitro.

#### 3.3.1. Cosmetic Ingredient

Vetiver oil has been claimed benefic in skin care, particularly for sensitive and older skin, due to its antiseptic, tonic and detoxifying properties [33]. Declared useful to balance sebaceous gland activity, it hence helps normalize oily skin and clearing acne [8]. It is also claimed to promote skin rejuvenation and to strengthen connective tissue, thus assisting with wound healing of mature, irritated and inflamed skins [12,33]. Vetiver oil is also known to replenish moisture in dehydrated and dry skins and even to prevent stretch marks [12]. Therefore, some authors report the use of vetiver oil in cosmetic formulas recommended for the treatment of skin’ overproduction of sebum, resulting in acne flare-ups and weeping sores [36]. Not toxic and nonirritant, vetiver oil also presents deodorizing properties [5]. Despite the interest for this grass essential oil, only few studies have been published to scientifically assess these cosmetic allegations and furthermore the published results are often contradictory. We then undertook a series of bioassays to clarify this situation and confirm or disconfirm these cosmetic allegations.

As shown in Table 2, vetiver EOs display only weak anti-oxidant activity. These results are consistent with the observations of some authors [37]. On the contrary, Kim et al. (2005) suggest that crude vetiver oil is competitive to well-known synthetic anti-oxidants, e.g., BHT (butylated hydroxytoluene) and α-tocopherol, and that this activity might be linked to its β-vetivenene, β-vetivone and α-vetivone content, all three displaying strong anti-oxidant activities when tested individually [38]. Several other studies confirmed that vetiver oil exhibits significant anti-oxidant activity [8,39]. A larger set of vetiver EOs should be assayed using several anti-oxidant tests (DPPH, ORAC, etc.) to solve this recurrent contradiction about the anti-oxidant activity of this EO.

Similarly, only weak or even no whitening activity of these EOs was evidenced, an observation already mentioned in the literature [37]. While reporting the antimelanogenic activity of vetiver oil, Peng, et al. (2014) demonstrated that this activity is implemented through the downregulation of both the activity and the protein level of tyrosinase, the latter one being the principal target of the EO [8].

Vetiver oil displays some interesting lipoxygenase inhibitory activity, as already noticed in the literature, even it has never been used in aromatherapy for this potential [40]. One could imagine the creation of vetiver oil-based ingredients that would therefore have the dual function of perfuming and of skin soothing *via* this anti-inflammatory potency.

As presented in Table 2, vetiver oil displays no interesting anti-aging activity, as no inhibitory activity of collagenase or hyaluronidase was observed and only a weak anti-elastasic activity was evidenced; no mention of such an inhibitory activity could be found in the literature.

#### 3.3.2. Antimicrobial Agent/Cosmetic Preservative

Several studies have reported that vetiver oil possesses antibacterial and antifungal activities against various pathogenic strains. These activities have been assessed using different in vitro methods, such as the cup bore method, the disk diffusion method and more rarely, the broth dilution method: results were expressed either in %*v*/*v*, µL/mL or µg/mL, and were sometimes divergent [12,41]. Moreover, activities of vetiver extracts were also reported against several drug-resistant bacterial pathogeneses, but only few of these claims were actually confirmed by laboratory assays [42,43]. To confirm/disconfirm those reported results, in vitro antibacterial and antifungal activities of the commercial vetiver EOs analyzed in the present article were assessed using the microbroth dilution method against twenty bacterial pathogenic strains and two *Candida* species: the results, expressed as MICs, are presented in Table 3.

The commercial vetiver EOs appeared to be mostly active against Gram-positive strains, i.e., *Staphylococcus aureus* (both susceptible and resistant to methicillin), *Corynebacterium striatum* and against both *Bacillus* strains, with MICs comprised between 500 and 2000 µg/mL, (i.e., between 0.5 and 2 µL/mL or 0.05 to 0.2% *v*/*v*). One can observe a consensus in the reported literature: the authors report the antibacterial activity of vetiver oil against Gram-positive strain, mainly *S. aureus*, while no activity is mentioned against Gram-negative bacteria [44,45].

Hence, comparing the values presented in Table 3 with previously published ones, one can state that, although the method employed to assess these antibacterial activities is different, these results are in total accordance with those from the literature [45]. On the other hand, the MICs presented here are weaker than those previously reported for *S. aureus* [46]. Nevertheless, the interesting growth inhibition activity of vetiver EOs obtained on SARM strains must be noticed; resistance developed towards antibiotics by pathogenic strains is more and more frequently observed. This phenomenon may be linked to the massive use of conventional preservatives or therapeutic agents and this subsequent resistance developed by bacterial strains constitutes a real public health issue [47,48,49,50]. These engaging results against SARM pave the way for a new potential use of vetiver EOs as promising anti-SARM agents.

The antifungal MIC reported here are slightly higher than the published values [44,46]. Assessing the anticandidal activity of plant extracts, some researchers only consider activities equivalent to those of antifungal drugs, whereas others consider the higher values obtained for plant constituents [51], as licensed drugs are generally effective at considerably lower concentrations [52]. Consequently, a classification of plant materials was established based on their fungal inhibition capacity outlined by MIC values: strong inhibitors are characterized by MICs up to 500 µg/mL and are differentiated from moderate ones exhibiting MICs comprised between 600 and 1500 µg/mL and from weak ones presenting MICs higher than 1600 µg/mL [53]. Therefore, following this classification, vetiver EO appears to be a moderate inhibitor of *C. albicans* and a strong inhibitor of *C. glabrata*. This result is particularly outstanding: indeed, the incidence of systemic infections due to non-*albicans Candida* species has increased markedly since the 1970s [54,55,56], mainly due to the extensive use of prophylactic and therapeutic antifungals, and to the primary low susceptibility of some of these yeast species to azoles, i.e., most widely used antifungals [57]. For instance, in some countries such as the USA, *C. glabrata* is currently the second yeast species responsible for clinical forms of candidiasis [58,59]. Exhibiting a low primary susceptibility to azole drugs, this species also displays a high frequency of acquired resistance [60].

## 4. Discussion and Conclusion

Vetiver essential oil, key ingredient for the perfume/personal care industry, might be threatened by obsolescence—similar to many others flagship cosmetic ingredients—due to constant and rapid changes in personal tastes. Being the cornerstone of the economy of some vetiver EO-producing countries, new dissemination pathways of this grass EO were investigated in this article.

Based on a thorough literature survey, vetiver EO appeared as benefic in skin care but not all cosmetic allegations were scientifically certified, or, if so, the results were often contradictory, thus a series of bioassays was undertaken to clarify this situation. No convincing cosmetic activity could be raised.

Some antimicrobial assays have then been performed, as essential oils might be sources of interesting antibacterial/antifungal molecules [48]. The antibacterial activities reported were in relative accordance with those from the literature: vetiver EO is mainly active on Gram-positive strains, especially on *Staphylococcus aureus* strains, either susceptible or resistant to methicillin.

Recently, evaluating the ability of several EOs to remove *Staphylococcus aureus*, a food-borne pathogen, from food-processing facilities, Vázquez Sánchez et al. (2015) observed a significant reduction of the number of viable biofilm cells induced by almost all of the EOs tested, but none of them could inhibit completely the formation of those biofilms [61]. Essential oil-based treatments consisting in combinations of various EOs alone (in rotation) or together with other biocides might be conceived to prevent the appearance of resistant bacterial strains in the food industry. Besides, one should also note that the original antifungal activity already known against *C. albicans*, actually extends to *C. glabrata.* To our knowledge, no previous mention of such an activity of vetiver oil against *C. glabrata* could be identified in the literature. Vetiver EO might constitute a potential source of novel anti-*Candida* compounds and lead to the development of alternative antifungal agents.

Therefore, from our results as well as from previously published ones, it can be concluded that vetiver essential oil should be considered as a potential alternative for synthetic biocides against emerging antibiotic-resistant microorganisms, presenting less harmful side effects and lower treatment costs. None of the compounds characterized in the vetiver EOs analyzed has previously been identified as displaying any antibacterial or antifungal activity. Hence, the antimicrobial results obtained for vetiver EOs may tentatively be attributed to the joint action of their major components, but synergetic/antagonist effects of some less abundant constituents cannot be ruled out. A subsequent bio-guided fractionation of these essential oils should be considered to further identify specifically active compounds.

## Figures and Tables

**Table 1:** Composition of commercial vetiver essential oils.

Constituent	RI ^a,b^ HP-1/RI Lit	Vetiver EOs	Identification Method
n.i.	1360/-	0.4–1.0	
α-cubebene	1366/1355	tr–0.1	LRI, MS
α-ylangene	1370/1376	tr–0.2	LRI, MS
α-cedrene	1410/1418	0.1–0.3	LRI, MS
acoradi-2,4-ene	1421/1421	0.1–0.3	LRI
n.i.	1427/-	0.1–0.4	
n.i.	1443/-	0.3–0.8	
preziza-7(15)-ene	1448/1448	0.5–1.1	LRI
ziza-6(13)-ene = khusimene	1452/1453	0.1–0.3	LRI
n.i.	1462/-	0.1–0.4	
n.i.	1466/-	0.2–0.4	
α-amorphene	1475/1477	0.5–1.9	LRI, MS
n.i.	1481/-	1.0–2.2	
n.i.	1485/-	0.6–1.8	
γ-muurolene	1488/1474	0.2–0.6	LRI, MS
n.i.	1501/-	0.3–0.8	
δ-cadinene	1522/1520	0.9–1.3	LRI
α-calacorene	1529/1527	0.4–0.7	LRI, MS
β-vetivenene	1547/1547	/–4.6	LRI
n.i.	1554/-	1.1–1.3	
khusimone	1577/1577	1.2–2.3	LRI
khusian-2-ol	1666/1668	1.8–2.3	LRI, MS
13-nor-eudesma-4,6-dien-11-one	1685/1692	0.7–0.9	LRI, MS
eudesma-3,5-dien-1α-ol	1707/1708	1.7–1.8	LRI
khusimol	1730/1726	0.6–8.9	LRI
n.i.	1774/-	2.1–2.9	
β-vetivone	1785/1788	2.2–3.7	LRI, MS
α-vetivone = isonootkatone	1808/1813	8.4–13.3	LRI, MS
ziza-6(13)-en-12-yl acetate = khusimyl acetate	1832/1828	0.4–0.7	LRI, MS

Notes: ^a^ Compounds are listed in order of their elution time from a HP-1 column. Compositional values inferior to 0.1% are noted as traces (tr). Presence of a compound is indicated by its GC-FID percentage, absence is indicated by “/”. ^b^ RI = retention indices are determined on a HP-1 column using the homologous series of n-alkanes (C6-C24).

**Table 2:** In vitro cosmetic bioassays performed using three commercial vetiver essential oils.

Sample Reference	Anti-Oxidant Activity	Whitening Activity		Anti-Inflammatory Activity	Anti-Elastase Activity	Anti-Collagenase Activity	Anti-Hyaluronidase Activity
		L-Tyrosine	L-DOPA				
***Vy1501***	+	+	−	++	+	−	−
***Vy1502***	+	+	−	+	+	−	−
***Vy1503***	+	−	−	+	+	−	−

(−): no activity; (+): 0% < inhibition < 30%; (++): 30% < inhibition < 60%; (+++): 60% < inhibition < 90%; (++++): inhibition > 90%.

**Table 3:** Antimicrobial activities of vetiver EOs expressed as MICs on eight Gram-positive and 12 Gram-negative bacterial strains (µg/mL), and on two *Candida* species (µg/mL).

Bacterial Strains Tested	Ve1501	Ve1502	Ve1503
	MICs (µg/mL)		
***Gram-negative bacteria***			
*Acinetobacter baumannii* RCH	>2000	>2000	>1000
*Acinetobacter baumannii* SAN008	>2000	>2000	>1000
*Acinetobacter baumannii* AYE	>2000	>2000	>1000
*Escherichia coli* ATCC25922	>2000	>2000	>1000
*Escherichia coli* BLSE 15509082801	>2000	>2000	>1000
*Enterobacter aerogenes* 15509970101	>2000	>2000	>1000
*Enterobacter aerogenes* 15501261101	>2000	>2000	>1000
*Klebsiella pneumoniae* BHR (OXA48)	>2000	>2000	>1000
*Klebsiella pneumoniae* 15000077501	>2000	>2000	>1000
*Pseudomonas aeruginosa* ATCC27853	>2000	>2000	>1000
*Pseudomonas aeruginosa* 155089996501	>2000	>2000	>1000
*Pseudomonas aeruginosa* 15509942001	>2000	>2000	>1000
***Gram-positive bacteria***			
*SARM* 15004850001	1000	2000	≤1000
*SARM* 15004306601	≤500	2000	≤1000
*SASM* 15509530101	1000	2000	≤1000
*SASM* 15004159801	≤500	2000	≤1000
*SERM* 15511909903	1000	2000	≤1000
*Corynebacterium striatum* 12572545501	≤500	2000	≤1000
*Bacillus* sp. 15003287301	≤500	2000	≤1000
*Bacillus subtilis* 15000964701	≤500	2000	≤1000
**Fungal Strains Tested**	**MICs (µg/mL)**		
*Candida albicans* ATCC66396 (Amphotericin B-MIC_80_: 0.125 µg/mL)	800	800	200
*Candida glabrata* LMA901085 (Amphotericin B-MIC_80_: 0.5 µg/mL)	400	200	100

SARM: *Staphylococcus aureus* resistant to methicillin; SASM: *Staphylococcus aureus* susceptible to methicillin; SERM: *Staphylococcus epidermidis* resistant to methicillin. Amphotericin: antifungal agent used as positive control.

## References

[1] Rao R.R., Suseela M.R. (2000). *Vetiveria zizanioides* (Linn.) Nash—A multipurpose eco-friendly grass of India. Proc. Second Int. Conf. Vetiver Off. R. Dev. Proj. Board Bangk..

[2] Maffei M. (2002). Introduction to the Genus *Vetiveria*. Vetiveria. The Genus Vetiveria; Medicinal and Aromatic Plants-Industrial Profiles.

[3] Lawrence B.M. (2016). Progress in essential oils. Perfum. Flavorist.

[4] Wong W. (1976). Some folk medicinal plants from Trinidad. Econ. Bot..

[5] Chomchalow N., Chapman K. Other uses and utilization of vetiver. Proceedings of the 3rd Conference of Vetiver and Exhibition.

[6] Duke J.A., du Cellier J.L. (1993). Handbook of Alternative Cash Crops.

[7] Slinger V. (1997). Spreading the slips of vetiver grass technology: A lesson in technology diffusion from Latin America. TVN Newsl..

[8] Peng H.-Y., Lai C.-C., Lin C.-C., Chou S.-T. (2014). Effect of *Vetiveria zizanioides* essential oil on melanogenesis in melanoma cells: Downregulation of tyrosinase expression and suppression of oxidative stress. Sci. World J..

[9] Chiu K.K., Ye Z.H., Wong M.H. (2006). Growth of *Vetiveria zizanioides* and *Phragmities australis* on Pb/Zn and Cu mine tailings amended with manure compost and sewage sludge: A greenhouse study. Bioresour. Technol..

[10] Sreenath H.L., Jagadishchandra K.S., Bajaj Y.P.S. (1994). Vetiveria zizanioides (L.) Nash (Vetiver Grass): In vitro culture, regeneration, and the production of essential oils. Medicinal and Aromatic Plants VI.

[11] Filippi J.-J., Belhassen E., Baldovini N., Brevard H., Meierhenrich U.J. (2013). Qualitative and quantitative analysis of vetiver essential oils by comprehensive two-dimensional gas chromatography and comprehensive two-dimensional gas chromatography/mass spectrometry. J. Chromatogr. A.

[12] Chahal K.K., Bhardwaj U., Kaushal S., Sandhu A.K. (2015). Chemical composition and biological properties of *Chrysopogon zizanioides* (L.) Roberty syn. *Vetiveria zizanioides* (L.) Nash-A Review. Indian J. Nat. Prod. Resour..

[13] Weyerstahl P., Marschall H., Splittgerber U., Wolf D. (2000). 1, 7-Cyclogermacra-1 (10), 4-dien-15-al, a sesquiterpene with a novel skeleton, and other sesquiterpenes from Haitian vetiver oil. Flavour Fragr. J..

[14] Lavania U.C. Other uses and utilization of vetiver: Vetiver oil. Proceedings of the Third International Conference on Vetiver and Exhibition.

[15] Oscar T. Haïti: Premier Exportateur Mondial de Vétiver. https://challengesnews.com/haiti-premier-exportateur-mondial-de-vetiver/.

[16] McLafferty F.W., Stauffer D.B. (1989). The Wiley/NBS Registry of Mass Spectral Data.

[17] Boelens Aroma Chemical Information Service (1999). BACIS ESO 2000, the Complete Database of Essential Oils.

[18] Weyerstahl P., Marschall H., Splittgerber U., Wolf D., Surburg H. (2000). Constituents of Haitian vetiver oil. Flavour Fragr. J..

[19] Brand-Williams W., Cuvelier M.E., Berset C. (1995). Use of a free radical method to evaluate antioxidant activity. LWT Food Sci. Technol..

[20] Koleva I.I., van Beek T.A., Linssen J.P.H., de Groot A., Evstatieva L.N. (2002). Screening of plant extracts for antioxidant activity: A comparative study on three testing methods. Phytochem. Anal. PCA.

[21] Burger P., Landreau A., Azoulay S., Michel T., Fernandez X. (2016). Skin whitening cosmetics: Feedback and challenges in the development of natural skin lighteners. Cosmetics.

[22] Lu W., Zhao X., Xu Z., Dong N., Zou S., Shen X., Huang J. (2013). Development of a new colorimetric assay for lipoxygenase activity. Anal. Biochem..

[23] Debelle L., Tamburro A.M. (1999). Elastin: Molecular description and function. Int. J. Biochem. Cell Biol..

[24] Papakonstantinou E., Roth M., Karakiulakis G. (2012). Hyaluronic acid: A key molecule in skin aging. Dermato-Endocrinol.

[25] Baumann L. (2007). Skin ageing and its treatment. J. Pathol..

[26] Roy A., Sahu R.K., Matlam M., Deshmukh V.K., Dwivedi J., Jha A.K. (2013). In vitro techniques to assess the proficiency of skin care cosmetic formulations. Pharmacogn. Rev..

[27] National Committee for Clinical Laboratory Standards (2002). Reference Method for Broth Dilution Antifungal Susceptibility Testing of Yeasts: Approved Standard.

[28] Société Française de Microbiologie. http://www.sfm-microbiologie.org/.

[29] Société Française de Microbiologie, European Committee on Antimicrobial Susceptibility Testing Comité de l’antibiogramme de la Société Française de Microbiologie—Recommandations 2015 V.2.0 Juillet 2015. http://www.sfm-microbiologie.org/UserFiles/files/casfm/CASFMV2_030915.pdf.

[30] Belhassen E., Baldovini N., Brevard H., Meierhenrich U.J., Filippi J.-J. (2014). Unravelling the scent of vetiver: Identification of character-impact compounds. Chem. Biodivers..

[31] Lawrence B.M. (2008). Progress in essential oils. Perfum. Flavorist.

[32] Champagnat P., Figueredo G., Chalchat J.-C., Carnat A.-P., Bessiere J.-M. (2006). A study on the composition of commercial *Vetiveria zizanioides* oils from different geographical origins. J. Essent. Oil Res..

[33] Chomchalow N. (2001). The Utilization of Vetiver as Medicinal and Aromatic Plants with Special Reference to Thailand.

[34] Andersen N.H. (1970). The structures of zizanol and vetiselinenol. Tetrahedron Lett..

[35] Belhassen E., Filippi J.-J., Brévard H., Joulain D., Baldovini N. (2015). Volatile constituents of vetiver: A review. Flavour Fragr. J..

[36] Curtis S. (1996). Essential Oils.

[37] Manosroi A., Manosroi J. (2005). Free radical scavenging and tyrosinase inhibition activity of aromatic volatile oil from Thai medicinal plants for cosmetic uses. Acta Hortic..

[38] Kim H.-J., Chen F., Wang X., Chung H.Y., Jin Z. (2005). Evaluation of antioxidant activity of vetiver (*Vetiveria zizanioides* L.) oil and identification of its antioxidant constituents. J. Agric. Food Chem..

[39] Chou S.-T., Lai C.-P., Lin C.-C., Shih Y. (2012). Study of the chemical composition, antioxidant activity and anti-inflammatory activity of essential oil from *Vetiveria zizanioides*. Food Chem..

[40] Baylac S., Racine P. (2003). Inhibition of 5-lipoxygenase by essential oils and other natural fragrant extracts. Int. J. Aromather..

[41] Prabuseenivasan S., Jayakumar M., Ignacimuthu S. (2006). In vitro antibacterial activity of some plant essential oils. BMC Complement. Altern. Med..

[42] Singh P., Srinivasan K.K., Subburaju T., Singh S.K. (2011). Antifungal activity of alcoholic and aqueous extracts of *Vetiveria zizanioides*. J. Pharm. Res. Opin..

[43] Singh K.K., Maheshwari J.K. (1994). Traditional phytotherapy of some medicinal plants used by the Tharus of the Nainital District, Uttar Pradesh, India. Int. J. Pharmacogn..

[44] Hammer K.A., Carson C.F., Thormar M. (2011). Antibacterial and antifungal activities of essential oils. Lipids and Essential Oils as Antimicrobial Agents.

[45] Champagnat P., Sidibé L., Forestier C., Carnat A., Chalchat J.-C., Lamaison J.-L. (2007). Antimicrobial activity of essential oils from *Vetiveria nigritana* and *Vetiveria zizanioides* roots. J. Essent. Oil Bear. Plants.

[46] Hammer K.A., Carson C.F., Riley T.V. (1999). Antimicrobial activity of essential oils and other plant extracts. J. Appl. Microbiol..

[47] Yagupsky P. (2006). Selection of antibiotic-resistant pathogens in the community. Pediatr. Infect. Dis. J..

[48] Guinoiseau E. (2010). Molécules Antibactériennes Issues d’huiles Essentielles: Séparation, Identification et Mode d’action.

[49] Goossens H., Guillemot D., Ferech M., Schlemmer B., Costers M., van Breda M., Baker L.J., Cars O., Davey P.G. (2006). National campaigns to improve antibiotic use. Eur. J. Clin. Pharmacol..

[50] Alekshun M.N., Levy S.B. (2007). Molecular mechanisms of antibacterial multidrug resistance. Cell.

[51] Duarte M.C.T., Figueira G.M., Sartoratto A., Rehder V.L.G., Delarmelina C. (2005). Anti-*Candida* activity of Brazilian medicinal plants. J. Ethnopharmacol..

[52] Pauli A. (2006). Anticandidal low molecular compounds from higher plants with special reference to compounds from essential oils. Med. Res. Rev..

[53] Aligiannis N., Kalpoutzakis E., Mitaku S., Chinou I.B. (2001). Composition and antimicrobial activity of the essential oils of two *Origanum* species. J. Agric. Food Chem..

[54] Krcmery V., Barnes A.J. (2002). Non-*albicans Candida* spp. causing fungaemia: Pathogenicity and antifungal resistance. J. Hosp. Infect..

[55] Spampinato C., Leonardi D. (2013). *Candida* infections, causes, targets, and resistance mechanisms: Traditional and alternative antifungal agents. Biomed. Res. Int..

[56] De las Mercedes Oliva M., Gallucci M.N., Carezzano M.E., Demo M.S. (2013). Natural products as alternative treatments for *Candida* species resistant to conventional chemotherapeutics. Fighting Multidrug Resistance with Herbal Extracts, Essential Oils and Their Components.

[57] Brion L.P., Uko S.E., Goldman D.L. (2007). Risk of resistance associated with fluconazole prophylaxis: Systematic review. J. Infect..

[58] Trick W.E., Fridkin S.K., Edwards J.R., Hajjeh R.A., Gaynes R.P. (2002). Secular trend of hospital-acquired candidemia among intensive care unit patients in the United States during 1989–1999. Clin. Infect. Dis..

[59] Li L., Redding S., Dongari-Bagtzoglou A. (2007). *Candida glabrata*, an emerging oral opportunistic pathogen. J. Dent. Res..

[60] Vandeputte P., Pineau L., Larcher G., Noel T., Brèthes D., Chabasse D., Bouchara J.-P. (2011). Molecular mechanisms of resistance to 5-fluorocytosine in laboratory mutants of *Candida glabrata*. Mycopathologia.

[61] Vázquez-Sánchez D., Cabo M.L., Rodríguez-Herrera J.J. (2015). Antimicrobial activity of essential oils against *Staphylococcus aureus* biofilms. Food Sci. Technol. Int..

